# Independent and cumulative effects of resting heart rate and pulse pressure with type 2 diabetes mellitus in Chinese rural population

**DOI:** 10.1038/s41598-017-02758-1

**Published:** 2017-06-01

**Authors:** Panpan Wang, Yuqian Li, Xiaotian Liu, Quanxin Wang, Ying Guo, Yang Zhao, Linlin Li, Jingjing Fan, Hao Zhou, Zhenxing Mao, Gongyuan Zhang, Chongjian Wang

**Affiliations:** 1 Department of Epidemiology and Biostatistics, College of Public Health, Zhengzhou University, Zhengzhou, Henan PR China; 2 Department of Clinical Pharmacology, School of Pharmaceutical Science, Zhengzhou University, Zhengzhou, Henan PR China; 3 Department of Health Education, Yuzhou Center for Disease Control and Prevention, Xuchang, Henan PR China

## Abstract

The purpose was to explore the effects of resting heart rate (RHR) and pulse pressure (PP) independently as well as their cumulative effects on the risk of type 2 diabetes mellitus (T2DM) through cross-sectional study plus meta-analysis. A total of 8276 subjects aged 35–74 years from the Rural Diabetes, Obesity and Lifestyle (RuralDiab) study were included in the study. Meanwhile, two meta-analyses were conducted to validate the results of the epidemiological research. The results showed that RHR and PP were associated with higher risk of T2DM, and the corresponding adjusted *OR*(95%*CI*) for each quartiles were 1.00, 0.99(0.68–1.42), 1.58(1.13–2.20), 2.93(2.15–3.98) and 1.00, 1.06(0.75–1.48), 1.11(0.79–1.56), 1.45(1.03–2.03), respectively. The cumulative effect analysis indicated that the adjusted *OR*(*95%CI*) in the fast RHR-high PP were 3.36(2.26–4.99), 2.60(1.47–4.59), and 3.60(2.09–6.20) compared with the slow RHR-low PP for total population, male and female, respectively. Meta-analysis showed that the pooled effect values for RHR and PP were 1.94(1.64–2.31) and 1.25(1.04–1.51), respectively. This study demonstrated that elevated RHR and PP are independently associated with the risk of T2DM as well as the influences of conventional confounders, and fast RHR with high PP might cumulatively increase the risk of T2DM. However, the potential clinical application remains to be determined.

## Introduction

Type 2 Diabetes mellitus (T2DM) is one of the worldwide epidemic chronic non-communicational diseases bringing about heavy social-economic burden, and the age-standardized incidences of diabetes have been significantly increased since 1990^1^. A nationwide survey showed about 113.9 million adults with diabetes and 493.4 million with prediabetes^2^, which indicates that diabetes has became one of the greatest public health problems in China. The etiology of T2DM is complex and the pathogenesis has not been fully understood yet. Some risk factors such as obesity, family history of T2DM, low physical activity and other unhealthy lifestyles were related to T2DM^2, 3^. However, the onset process of T2DM is concealed and many patients are unaware until serious symptoms occurred, which might miss the best treatment stage. Thus, identification of risk factors of diabetes is crucial and urgent.

Resting heart rate (RHR) and pulse pressure (PP) are convenient measurements but important indicators of cardiovascular diseases^4, 5^. As a simple indicator of autonomic nervous system function, RHR varies with the activation or inactivation of sympathetic nerve^6^, and elevated RHR might be a risk factor associated with the development of diabetes, however, the results were inconsistent in different populations^7–14^. Previous studies explored the relationship between blood pressure and the risk of cardiovascular diseases, but relevant studies were usually focused on systolic blood pressure (SBP) and diastolic blood pressure (DBP), rather than PP^15–17^. As an important component of blood pressure as well as a parameter of arterial stiffness, PP was confirmed to be associated with cardiovascular events and mortality^18, 19^. Few epidemiological studies indicated that high PP might have an increased risk for the development of T2DM^20–22^. However, data from different population remained limited and the results were also inconsistent^23–25^. In addition, no study has been published in exploring whether resting heart rate and pulse pressure have cumulative effect on the risk of T2DM. More importantly, study emphasizes the association of RHR, PP and the risk of T2DM by combining epidemiological research and meta-analysis has not been reported yet. Therefore, the purpose of this study was to explore the separate relationship and cumulative effect of RHR and PP on the risk of T2DM in Chinese rural population combining cross-sectional study and meta-analysis.

## Results

### General characteristics

Table 1 shows the general characteristics of the participants. Among the 8276 participants, 383 subjects (156 male and 227 female) were identified with undiagnosed T2DM. For participants with undiagnosed T2DM, higher BMI, higher fasting plasma glucose (FPG) could be observed in both genders (*P* < 0.001). Moreover, subjects with undiagnosed T2DM were more likely to have faster RHR, higher SBP, DBP and PP (*P* < 0.001).

**Table 1:** General characteristics of the subjects (n = 8276).

Variables	Total (n = 8276)			Male (n = 2981)			Female (n = 5295)		
	Normal	Undiagnosed T2DM	*P*	Normal	Undiagnosed T2DM	*P*	Normal	Undiagnosed T2DM	*P*
Age (years), mean ± SD	54.71 ± 9.95	57.26 ± 8.89	<0.001	55.58 ± 10.37	55.93 ± 9.60	0.683	54.22 ± 9.67	58.17 ± 8.27	<0.001
Education, n (%)			0.059			0.607			0.001
≤Primary school	3140(39.81)	171(44.65)		851(30.15)	44(28.21)		2289(45.19)	127(55.95)	
≥Middle school	4748(60.19)	212(55.35)		1972(69.85)	112(71.79)		2776(54.81)	100(44.05)	
Marital status, n (%)			0.004			0.090			0.018
Married/cohabitation	7156(91.18)	332(86.91)		2585(92.19)	137(88.39)		4571(90.62)	195(85.90)	
Divorced/widowed/unmarried	692(8.82)	50(13.09)		219(7.81)	18(11.61)		473(9.38)	32(14.10)	
Physical activity, n (%)			0.002			0.112			0.007
Low	2725(34.53)	162(42.30)		1102(39.02)	74(47.43)		1623(32.02)	88(38.77)	
Moderate	1592(20.17)	81(21.15)		358(12.68)	17(10.90)		1234(24.35)	64(28.19)	
High	3575(45.30)	140(36.55)		1364(48.30)	65(41.67)		2211(43.63)	75(33.04)	
More vegetables & fruits intake, n (%)	1946(24.66)	81(21.15)	0.119	720(25.50)	27(17.31)	0.022	1226(24.19)	54(23.79)	0.890
High-fat diet, n (%)	1898(24.05)	93(24.28)	0.918	906(32.09)	53(33.97)	0.625	992(19.57)	40(17.62)	0.467
Current smoking, n (%)	1424(18.04)	65(16.97)	0.100	1415(50.11)	65(41.67)	0.116	9(0.18)	0(0.0)	0.182
Current drinking, n (%)	1353(17.14)	75(19.58)	0.217	1224(43.33)	68(43.59)	0.949	129(2.55)	7(3.08)	0.616
Family history of diabetes, n (%)	558(7.07)	34(8.88)	0.180	188(6.65)	22(14.10)	<0.001	370(7.30)	12(5.29)	0.251
BMI (kg/m^2^)	25.15 ± 3.39	26.60 ± 3.70	<0.001	24.79 ± 3.32	26.75 ± 3.75	<0.001	25.36 ± 3.41	26.50 ± 3.68	<0.001
FPG (mmol/l)	5.19 ± 0.60	9.17 ± 2.56	<0.001	5.18 ± 0.64	9.21 ± 2.49	<0.001	5.19 ± 0.58	9.15 ± 2.61	<0.001
Resting heart rate (beats/min)	76.41 ± 10.06	81.62 ± 12.24	<0.001	74.74 ± 10.10	80.46 ± 11.10	<0.001	77.33 ± 9.92	82.43 ± 12.92	<0.001
Systolic blood pressure (mmHg)	125.30 ± 19.07	131.27 ± 18.87	<0.001	126.69 ± 17.67	129.31 ± 17.11	0.071	124.52 ± 19.76	132.63 ± 19.91	<0.001
Diastolic blood pressure (mmHg)	78.68 ± 11.53	82.01 ± 11.10	<0.001	80.42 ± 11.57	83.22 ± 10.67	0.003	77.71 ± 11.40	81.17 ± 11.33	<0.001
Pulse pressure (mmHg)	46.62 ± 12.22	49.27 ± 13.00	<0.001	46.27 ± 10.97	46.09 ± 10.84	0.841	46.82 ± 12.87	51.45 ± 13.91	<0.001

BMI, body mass index; FPG, fasting plasma glucose.

### Independent effects of RHR/PP on T2DM

Table 2 summarizes the crude and adjusted *ORs* (95%*CI*) of RHR/PP with the risk of undiagnosed T2DM. The results showed that RHR was positively associated with the risk of undiagnosed T2DM, and the adjusted *ORs*(95%*CI*) were 1.00, 0.99(0.68–1.42), 1.58(1.13–2.20), and 2.93(2.15–3.98) in total population. Similarly relations were found for both genders. The risk of undiagnosed T2DM displayed increasing tendencies with RHR in both genders (*P*_*trend*_ < 0.001).

**Table 2:** Analyses for the risk of undiagnosed T2DM according to the resting heart rate and pulse pressure (n = 8276).

Gender	Variables	Normal n (%)	Undiagnosed T2DM n (%)	Crude *OR*(95%*CI*)	Adjusted *OR*(95%*CI*)^#^
Total	Resting heart rate (beats/min)				
	Q1: 40–69	1972(24.98)	63(16.45)	1.00	1.00
	Q2: 70–75	1926(24.40)	59(15.40)	0.96 (0.67–1.38)	0.99(0.68–1.42)
	Q3: 76–82	2061(26.11)	94(24.54)	1.43(1.03–1.98)	1.58(1.13–2.20)
	Q4: 83–136	1934(24.50)	167(43.60)	2.70(2.01–3.64)	2.93(2.15–3.98)
	*P*_*trend*_ for resting heart rate			0.000	0.000
	Pulse Pressure (mmHg)				
	Q1: 17–37	1815(22.99)	64(16.71)	1.00	1.00
	Q2: 38–44	2086(26.43)	84(21.93)	1.14(0.82–1.59)	1.06(0.75–1.48)
	Q3: 45–52	1911(24.21)	93(24.28)	1.38(0.99–1.91)	1.11(0.79–1.56)
	Q4: 53–117	2081(26.37)	142(37.08)	1.94(1.43–2.62)	1.45(1.03–2.03)
	*P*_*trend*_ for pulse pressure			0.000	0.022
Male	Resting heart rate (beats/min)				
	Q1: 41–67	659(23.33)	14(8.97)	1.00	1.00
	Q2: 68–73	727(25.73)	32(20.51)	2.07(1.10–3.92)	2.01(1.05–3.85)
	Q3: 74–80	712(25.20)	37(23.72)	2.45(1.31–4.57)	2.47(1.31–4.65)
	Q4: 81–136	727(25.73)	73(46.79)	4.73(2.64–8.45)	4.69(2.58–8.55)
	*P*_*trend*_ for resting heart rate			0.000	0.000
	Pulse Pressure (mmHg)				
	Q1: 20–38	657(23.26)	36(23.08)	1.00	1.00
	Q2: 39–44	731(25.88)	33(21.15)	0.82(0.51–1.34)	0.81(0.49–1.34)
	Q3: 45–51	712(25.20)	44(28.21)	1.13(0.72–1.77)	1.05(0.65–1.69)
	Q4: 52–110	725(25.66)	43(27.56)	1.08(0.69–1.71)	0.86(0.51–1.44)
	*P*_*trend*_ for pulse pressure			0.444	0.811
Female	Resting heart rate (beats/min)				
	Q1: 40–70	1278(25.22)	44(19.38)	1.00	1.00
	Q2: 71–76	1232(24.31)	31(13.66)	0.73(0.46–1.17)	0.74(0.46–1.19)
	Q3: 77–82	1203(23.74)	46(20.26)	1.11(0.73–1.69)	1.21(0.79–1.85)
	Q4: 83–124	1355(26.74)	106(46.70)	2.27(1.59–3.26)	2.45(1.70–3.53)
	*P*_*trend*_ for resting heart rate			0.000	0.000
	Pulse Pressure (mmHg)				
	Q1: 17–37	1268(25.02)	34(14.98)	1.00	1.00
	Q2: 38–44	1245(24.57)	45(19.82)	1.35(0.86–2.12)	1.21(0.76–1.92)
	Q3: 45–53	1228(24.23)	55(24.23)	1.67(1.08–2.58)	1.21(0.77–1.92)
	Q4: 54–117	1327(26.18)	93(40.97)	2.61(1.75–3.90)	1.69(1.07–2.67)
	*P*_*trend*_ for pulse pressure			0.000	0.021

^**#**^Adjusted for age (years), sex, smoking, drinking, education, physical activity, marital status, intakes of vegetables and fruits, high–fat diet, family history of diabetes and BMI (kg/m^2^).

Likewise, elevated PP was also associated with the risk of undiagnosed T2DM, and the adjusted *ORs*(95%*CI*) were 1.00, 1.06(0.75–1.48), 1.11(0.79–1.56), and 1.45(1.03–2.03) in total population. Similar association was found in female but not in male, and the adjusted *ORs*(95%*CI*) were 1.00, 1.21(0.76–1.92), 1.21(0.77–1.92), and 1.69(1.07–2.67) for the female. Increased risks of undiagnosed T2DM were displayed with PP in the total and female participants (*P*_*trend*_ = 0.022 for total, *P*_*trend*_ = 0.021 for the female).

### Cumulative effects of RHR/PP with T2DM

Table 3 presents the cumulative effect of RHR and PP on undiagnosed T2DM. Compared with the slow RHR-low PP group, the participants in other groups have higher risk of undiagnosed T2DM, and the adjusted *ORs*(95%*CI*) were 1.68(1.11–2.55), 2.90(1.95–4.32), and 3.36(2.26–4.99), respectively. Among the four groups based on RHR and PP (slow-low, slow-high, fast-low, and fast-high), the fast-high group was at the highest risk of undiagnosed T2DM. The similarly relation was found in both genders, and the corresponding *ORs*(95%CI) were 2.60(1.47–4.59) for male and 3.60(2.09–6.20) for female, respectively. However, there was no significant interaction between RHR and PP on the risk of undiagnosed T2DM no matter in the multiplicative interaction model or the additive model.

**Table 3:** Cumulative effect of slow/fast resting heart rate and low/high pulse pressure for undiagnosed T2DM (n = 8276).

Gender	Level^*^		Normal n (%)	Undiagnosed T2DM n (%)	Crude *OR (95%CI)*	Adjusted *OR (95%CI)* ^#^
	Resting heart rate	Pulse Pressure				
Total	Slow	Low	1735(21.98)	33(8.62)	1.00	1.00
		High	2163(27.40)	89(23.24)	2.16(1.44–3.24)	1.68(1.11–2.55)
	Fast	Low	2166(27.44)	115(30.03)	2.79(1.89–4.13)	2.90(1.95–4.32)
		High	1829(23.17)	146(38.12)	4.20(2.86–6.16)	3.36(2.26–4.99)
	*P*_*interaction*_ for resting heart rate and pulse pressure				0.135	0.129
Male	Slow	Low	632(22.37)	17(10.90)	1.00	1.00
		High	754(26.69)	29(18.59)	1.43(0.78–2.63)	1.17(0.62–2.20)
	Fast	Low	756(26.76)	52(33.33)	2.56(1.46–4.47)	2.43(1.38–4.30)
		High	683(24.18)	58(37.18)	3.16(1.82–5.48)	2.60(1.47–4.59)
	*P*_*interaction*_ for resting heart rate and pulse pressure				0.690	0.817
Female	Slow	Low	1122(22.14)	17(7.49)	1.00	1.00
		High	1388(27.39)	58(25.55)	2.76(1.60–4.76)	1.91(1.08–3.36)
	Fast	Low	1391(27.44)	62(27.31)	2.94(1.71–5.06)	3.03(1.75–5.23)
		High	1167(23.03)	90(39.65)	5.09(3.01–8.60)	3.60(2.09–6.20)
	*P*_*interaction*_ for resting heart rate and pulse pressure				0.153	0.150

*Slow and low are defined as ‘Q1 + Q2’, and fast and high are defined as ‘Q3 + Q4’.

#Adjusted for age (years), sex, smoking, drinking, education, physical activity, marital status, intakes of vegetables and fruits, high-fat diet, family history of diabetes and BMI (kg/m^2^).

### Meta-analysis

**Figure 1:**
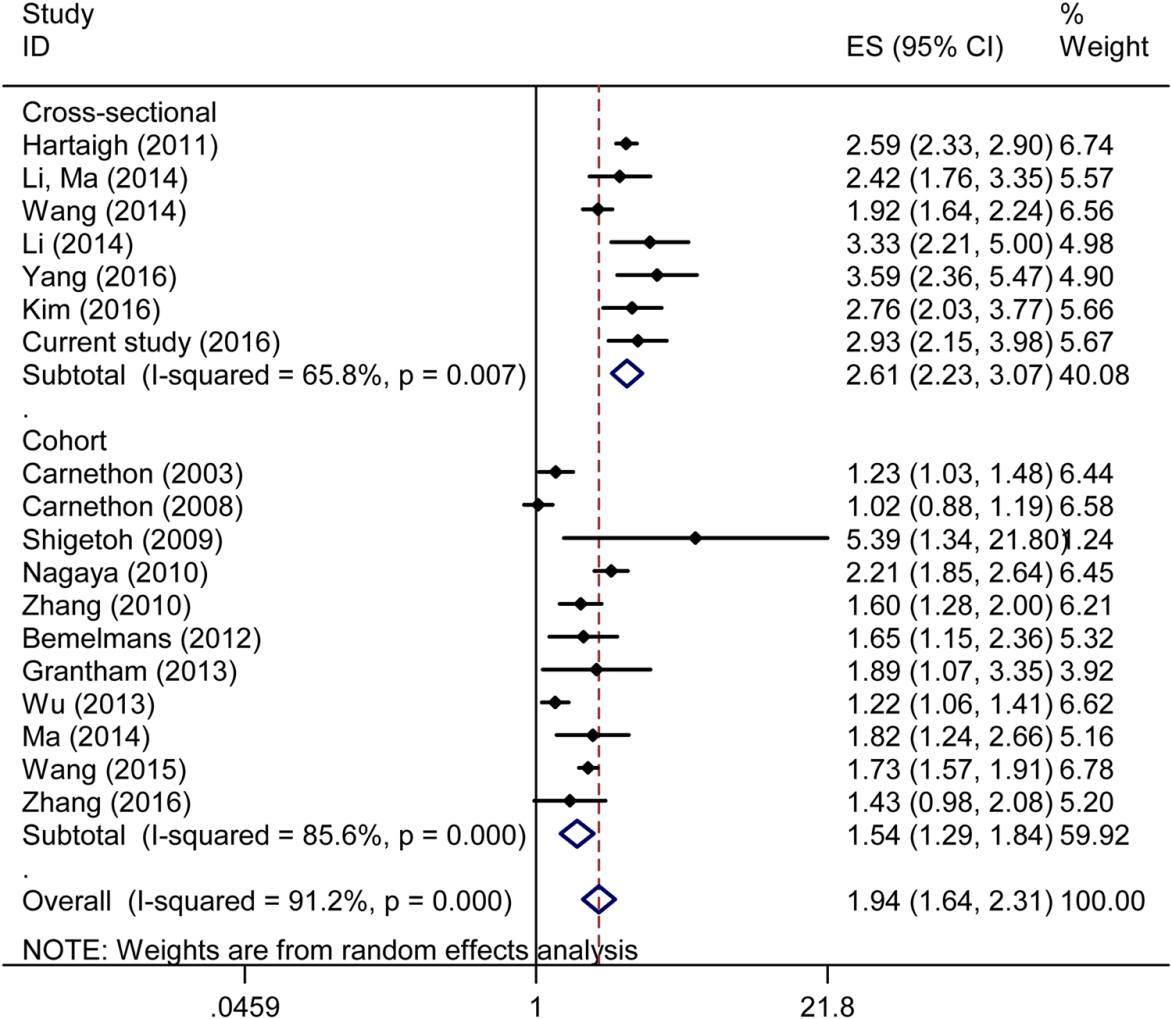
Forest plot of resting heart rate and diabetes risk for the selected studies.

For RHR, a total of eighteen studies were included in the meta-analysis^7–14, 26–34^. Figure 1 demonstrates the association between RHR and the risk of T2DM. The overall pooled effect size for highest *vs*. lowest RHR was 1.94(95%*CI*: 1.64–2.31). The subtotal pooled effect sizes were 2.61(95%*CI*: 2.23–3.07) for the cross-sectional studies and 1.54(95%*CI*: 1.29–1.84) for the cohort studies respectively. There was obvious heterogeneity across studies (*I* ^*2*^ > 50%). No evidence of publication bias was found through the *Begg’s* test (*P* = 0.256).

**Figure 2:**
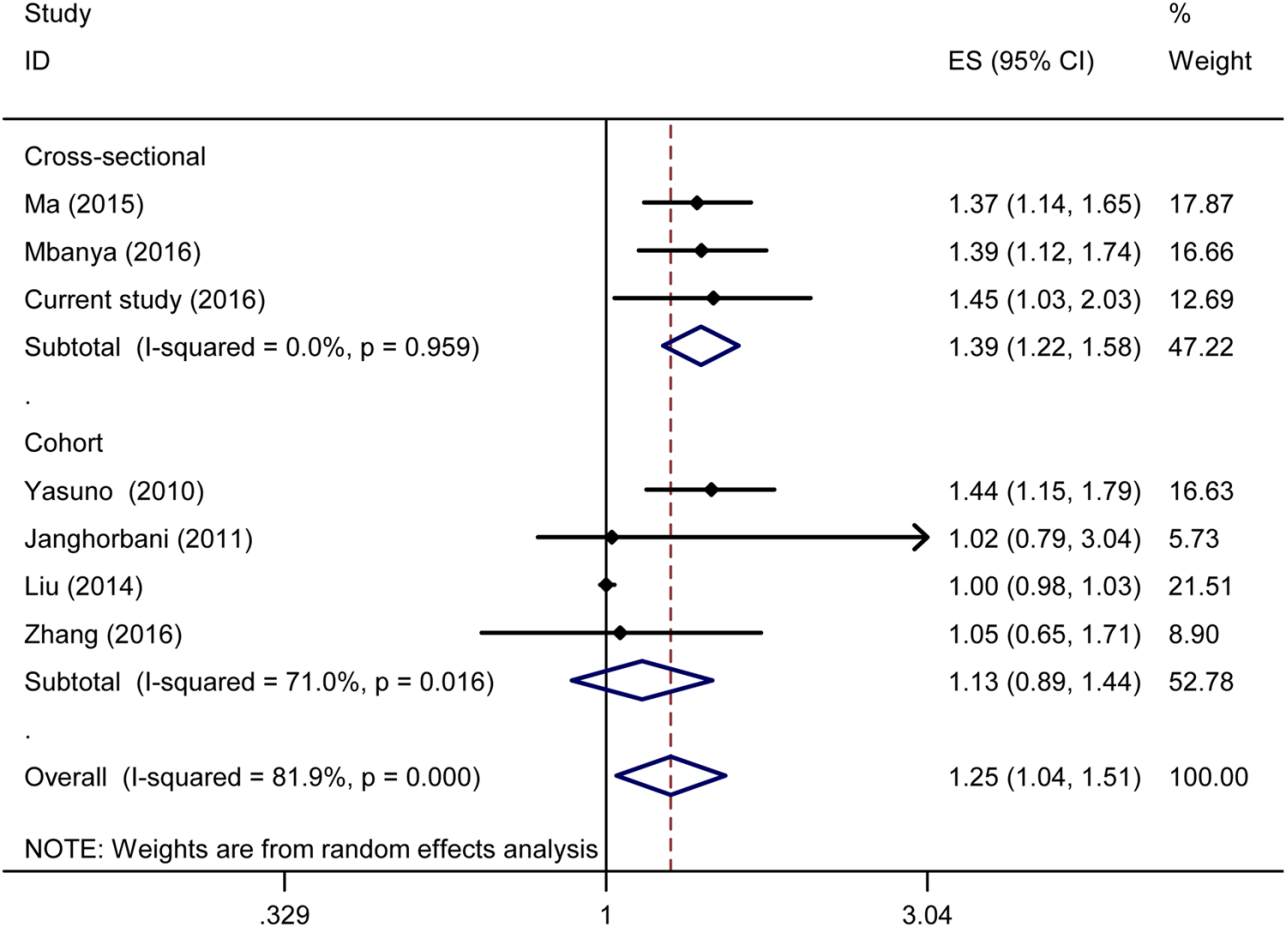
Forest plot of pulse pressure and diabetes risk for the selected studies.

For PP, seven studies were included in the final meta-analysis^20–25^. The relation between PP and the risk of T2DM is shown in Fig. 2. The overall pooled effect size for highest *vs*. lowest PP was 1.25(95%*CI*: 1.04–1.51). The subtotal pooled effect sizes were 1.39(95%*CI*: 1.22–1.58) and 1.13(95%*CI*: 0.89–1.44) for the cross-sectional and cohort studies, respectively. Unlike the cohort studies, no obvious heterogeneity was found in the cross-sectional studies. No evidence of publication bias was found through *Begg’s* test (*P* = 0.368).

## Discussion

To our knowledge, this is the first study combining cross-sectional survey and meta-analysis to explore the association between RHR, PP and the risk of T2DM. In this population-based study, the results showed that RHR and PP were positively associated with the risk of undiagnosed T2DM in China rural residents aged 35–74 years. Moreover, this study combined RHR and PP as a new variable to calculate the cumulative effect on risk of undiagnosed T2DM, and found that subjects with fast RHR and high PP were at the highest risk of undiagnosed T2DM in this rural population. The similar relationships were also found in the meta-analysis.

RHR and PP are simple indicators of autonomic nervous system function. Elevated RHR and/or PP might reflect the disorder of autonomic nervous system leading to the activation of the sympathetic nervous system^6^. Sympathetic over-activity is in connection with obesity, reduced insulin sensitivity, inflammation and the metabolic syndrome^35–38^, all of which could increase the risk of T2DM. Therefore, chronic sympathetic over-activity implies the development of T2DM in subjects with tachycardia and/or high PP. Moreover, subjects with diabetes tend to present a certain degree of the autonomic dysfunction leading to elevated RHR and/or PP^39–41^.

RHR has been identified as an independent risk factor of T2DM in previous studies^12–14^. In this study, compared to subjects with RHR ≤ 69 bpm, subjects with RHR ≥ 83 bpm were 2.93 times more likely to have undiagnosed T2DM. The present data showed that subjects with RHR in the highest group were 4.69 times for male, 2.45 times for female to have undiagnosed T2DM compared to the subjects with RHR in the lowest group. This difference might mostly due to the varied metabolic features in the two genders. In addition, compared with female, male tended to live with an unhealthy lifestyle, such as smoking, alcohol drinking, high fat intake and so on. All of these might lead to the gender difference of the results.

At present, researches concerning about the relationship between PP and diabetes risk were limited and controversial. PP was identified as a risk factor for new-onset diabetes in Japanese population^22^ whereas another study in Iran reported that PP was not associated with diabetes^23^. A study in Chengdu explored whether PP could be a predictor of diabetes in middle-aged subjects but got negative result^24^. A recent study from Chinese population revealed that subjects with PP ≥ 53 mmHg were 1.45 times chance to have undiagnosed T2DM^25^. The similar relationship could be seen in female (1.69 times) but not in the male in this study. The gender difference in risk of diabetes might due to the sexual hormone levels in women, especially for these after menopause.

The present study explored the separate relationship between RHR, PP and the risk of undiagnosed T2DM, which was consistent with the results of the meta-analysis. In addition, this study combined RHR and PP as a new variable to assess the cumulative effect on undiagnosed T2DM. Compared with slow RHR-low PP, subjects with fast RHR and high PP were 3.36 folds chance to have undiagnosed T2DM (2.60 folds for male, 3.60 folds for female). Fast RHR and high PP had the cumulative effect on the risk of T2DM. Thus, subjects with fast RHR-high PP might be more susceptible to T2DM.

Results from this research have essential meaning for diabetes prevention. Diabetes mellitus could bring about a series of complications, which badly influence the peoples’ life quality accompanied by heavy disease burden. This study highlights that fast resting heart rate and high pulse pressure are associated with diabetes risk. Therefore, the two convenient measurements are conducive to screen people with high diabetes risk, especially in condition limited areas. Effective measures to control resting heart rate or pulse pressure could prevent or delay the occurrence of diabetes.

Although this is the first study to estimate the cumulative effect of RHR, PP on risk of T2DM, several limitations need to be noticed when intercepting the results. Firstly, the diagnosis of T2DM was mainly based on FPG, absence of oral glucose tolerance test (OGTT) and detection of glycosylated hemoglobin, which might misestimate the number of the patients. However, subgroup analysis of the meta-analysis showed that using FPG as a diagnosis criterion to identify T2DM got similar results compared with OGTT/HbA1c (Supplementary Figure S3 and Supplementary Figure S4). Secondly, the subjects were only from one province, which may not well represent the whole rural areas of China. However, the rural population of Henan province accounted for 10% of China’s rural population. Therefore, results from this study could represent the relationship of resting heart rate, pulse pressure and T2DM to some extent. Thirdly, some important covariates, such as the data regarding dietary intake and lifestyle might have reporting bias, but potential covariates were adjusted as much as possible. Fourthly, the results were based on a cross-sectional design and materials collected could only reflect the exposure or disease status at that permanent time, which could not draw a causal effect conclusion. Therefore, the prospective cohort study is needed to establish. In addition, meta-analysis showed that the adverse effects of elevated RHR and PP on T2DM risk were more obvious in the cross-sectional study than the cohort study. This implied that our study might overestimate the association between RHR, PP and diabetes risk in some degree. Despite these limitations, the results based on the synthesizing cross-sectional study and meta-analysis could represent the relationship between RHR, PP and the risk of T2DM as well as the cumulative effect.

In conclusion, this study demonstrates that RHR and PP are positively associated with the risk of undiagnosed T2DM in Chinese rural residents aged 35–74 years. In addition, fast resting heart rate and high pulse pressure were related to higher risk of undiagnosed T2DM. All of these provide some supports for taking RHR and PP as two risk markers in T2DM prediction. However, the multi-centered long term follow-up studies are needed to further confirm the assumption.

## Methods

### Study subjects

The subjects were from the Rural Diabetes, Obesity and Lifestyle (RuralDiab) study which conducted in rural areas of Henan Province in China. From July 2013 to August 2015, a total of 12 602 residents from Yuzhou, Wuzhi, and Houzhai county took part in the survey and completed the standardized questionnaires by trained interviewers, of which, 11135 subjects aged between 35 to 74 years old. Candidates were defined as ineligible if they were: 1) with known diabetes (n = 999); 2) with severe physical or psychological diseases (n = 1715); 3) with tuberculosis, hepatitis, or other infectious diseases (n = 118); 4) missing information on RHR, PP or blood glucose (n = 27). Following these criteria, 8276 eligible subjects (2981 male and 5295 female) were included in the present study.

The present study was conducted according to the Declaration of Helsinki. The procedure was approved by the Zhengzhou University Life Science Ethics Committee, and written informed consent was obtained from all the participants of the study.

### Data collection and laboratory measurement

Data were collected through face to face interview using a standardized questionnaire which included the information of demographic characteristics, socioeconomic status, family and individual disease history, dietary intake and lifestyles. Briefly, the education level was categorized into primary school or below, middle school or above. Marital status was classified into married/cohabitation, unmarried/divorced/widowed. Current smoking was defined as consuming 1 cigarette/day or more for at least 6 months. Current drinking was defined as having alcohol drink for at least 12 times a year. According to the Chinese Nutrition Society^42^, more vegetables and fruits intake were defined as consuming an average of more than 500 g vegetables and fruits per day, and high fat intake was defined as consuming an average of more than 75 g fat per day. Physical activity was classified into three levels (low, moderate and high) according to the International Physical Activity Questionnaire (IPAQ)^43^.

Anthropometric parameters were measured twice and the average readings were taken for statistic analysis. Body weight and height were measured in light clothing without shoes to the nearest 0.1 kg and 0.1 cm, respectively. Body mass index (BMI) was calculated as weight in kilograms divided by the square of height in meters.

After overnight fasting (at least 8 hours), venous blood was drawn by clinical physicians in the vacuum tubes with ethylenediaminetetraacetic acid dipotassium salt (EDTA-K2). Blood samples were centrifuged for 10 minutes at 3000 rpm, and then the plasma samples were sent to measure biochemical indicators with cold-chain transportation. FPG was measured within 8 hours by Roche Cobas C501 automatic biochemical analyzer with glucose oxidative method (GOD-PAP).

### Assessment of resting heart rate and pulse pressure

Blood pressure and RHR were measured by using electronic sphygmomanometer (Omron HEM-7071A, Japan), and PP was calculated by SBP subtracting DBP. According to the America Heart Association’s standardized protocol^44^, the subjects were required to sit at least 5 minutes and blood pressure were measured for three times. Average of the three readings was taken for the analysis. If a difference more than 5 mmHg or 5 beats/min was observed, the closest two values would be reserved.

### Assessment of outcomes

According to the American Diabetes Association (ADA) (2009) guideline^45^, participants were defined as diabetes mellitus if FPG ≥7.0 mmol/L and/or self-reported current treatment with insulin or oral hypoglycemic agents. Undiagnosed diabetes mellitus was defined as having FPG ≥7.0 mmol/L excluding self-reported current treatment with anti-diabetes medication among those with diabetes. In addition, we excluded type 1 diabetes mellitus, gestational diabetes mellitus and diabetes due to other causes.

### Meta-analysis

Two meta-analyses containing previous published studies and the current study were conducted to demonstrate the association of RHR and PP with the risk of T2DM. The relevant papers were searched published before October 31, 2016 in the PubMed, Web of science, EMBASE, China National Knowledge Infrastructure (CNKI), Wanfang database. (“Resting heart rate” OR “heart rate” OR “pulse rate”) AND (“diabetes” OR “diabetes mellitus” OR “type 2 diabetes mellitus”), (“Pulse pressure” OR “Blood pressure”) AND (“diabetes” or “diabetes mellitus” OR “type 2 diabetes mellitus”) were used as the searching terms, respectively. The inclusion criteria for the study were as follows: (1) had explored the association between RHR/PP and T2DM by cross sectional or cohort study; (2) had provided the relative risk data, such as odds ratios, risk ratios or hazard ratios. We also checked the reference lists concerning this topic to identify more available studies. The flow chart of study selection process was described in Supplementary Figure S1 and Supplementary Figure S2, respectively. Data extraction was conducted by two reviewers independently, and the disagreements were discussed with a third reviewer. The extracted information was as follows: author, publication year, country of study conducted, the project title, follow-up duration, sample size, age range, covariates, diagnostic method, number of cases, RHR/PP level, effect size and 95%*CI* for the association. The details were shown in Supplementary Table S1 and Supplementary Table S2.

### Statistical analysis

The statistical analysis of the survey data was performed using SAS9.3 software package (SAS Institute, USA) and *P* < 0.05 was considered to be statistically significant. All the data were analyzed by gender. For continuous and categorical variables, *t* test and chi-square test were performed to identify the difference of characteristics, respectively. RHR and PP were stratified into four levels according to quartiles, respectively. Logistic regression models were built to estimate the crude and adjusted odds ratio (*OR*) and 95% confidence interval (*CI*) for the risk of T2DM. Besides, trend chi-square test was conducted to evaluate the tendency of T2DM risk with RHR or PP. In order to evaluate the cumulative effect of RHR and PP on the risk of T2DM, RHR and PP were then categorized into two levels: slow/low (Q1 + Q2) and fast/high (Q3 + Q4). Thus, we got four groups: slow-low (reference), slow-high, fast-low, and fast-high. A multiplicative interaction model and an additive model were set up to quantify the interaction effect on risk of T2DM.The adjusted variables included age, sex, smoking, alcohol drinking, education, physical activity, marital status, intakes of vegetables and fruits, high-fat diet, family history of diabetes, and BMI.

The meta-analyses were performed using the STATA software package, V.11.0 (STATACorp, College Station, Texas, USA). Study heterogeneity was estimated by the *I* ^*2*^ statistic and a random-effect model was used to calculate the pooled estimates of T2DM risk and the corresponding 95% *CI*. Begg’s test was applied to evaluate the potential publication bias.

## Electronic supplementary material

Supplementary Information
