# Supplementary material for: Independent and cumulative effects of resting heart rate and pulse pressure with type 2 diabetes mellitus in Chinese rural population
Source: Sci Rep. 2017 Jun 1;7:2625. doi: 10.1038/s41598-017-02758-1 (PMC5454018; doi:10.1038/s41598-017-02758-1)
Supplement: Supplementary file 1 — Supplementary Information [file 41598_2017_2758_MOESM1_ESM.pdf]

Title:

Independent and cumulative effects of resting heart rate and pulse pressure with type 2 diabetes mellitus in Chinese rural population

Short title:

Heart rate, pulse pressure and diabetes

Author:

Panpan Wang[1†], Yuqian Li[2†], Xiaotian Liu[1], Quanxin Wang[3], Ying Guo[3], Yang Zhao[1], Linlin Li[1], Jingjing Fan[1], Hao Zhou[1], Zhenxing Mao[1], Gongyuan Zhang[1], Chongjian Wang[1*]

Authors affiliations:

[1] Department of Epidemiology and Biostatistics, College of Public Health, Zhengzhou University, Zhengzhou, Henan, *PR* China.

[2] Department of Clinical Pharmacology, School of Pharmaceutical Science, Zhengzhou University, Zhengzhou, Henan, *PR* China.

[3] Department of Health Education, Yuzhou Center for Disease Control and Prevention, Xuchang, Henan, PR China.

[†] Contributed equally to this work.

[*] **Correspondence author**

Dr. Chongjian Wang

Department of Epidemiology and Biostatistics

College of Public Health, Zhengzhou University

100 Kexue Avenue, Zhengzhou, 450001, Henan, *PR* China

Phone: +86 371 67781452

Fax: +86 371 67781919

E-mail: tjwcj2005@126.com

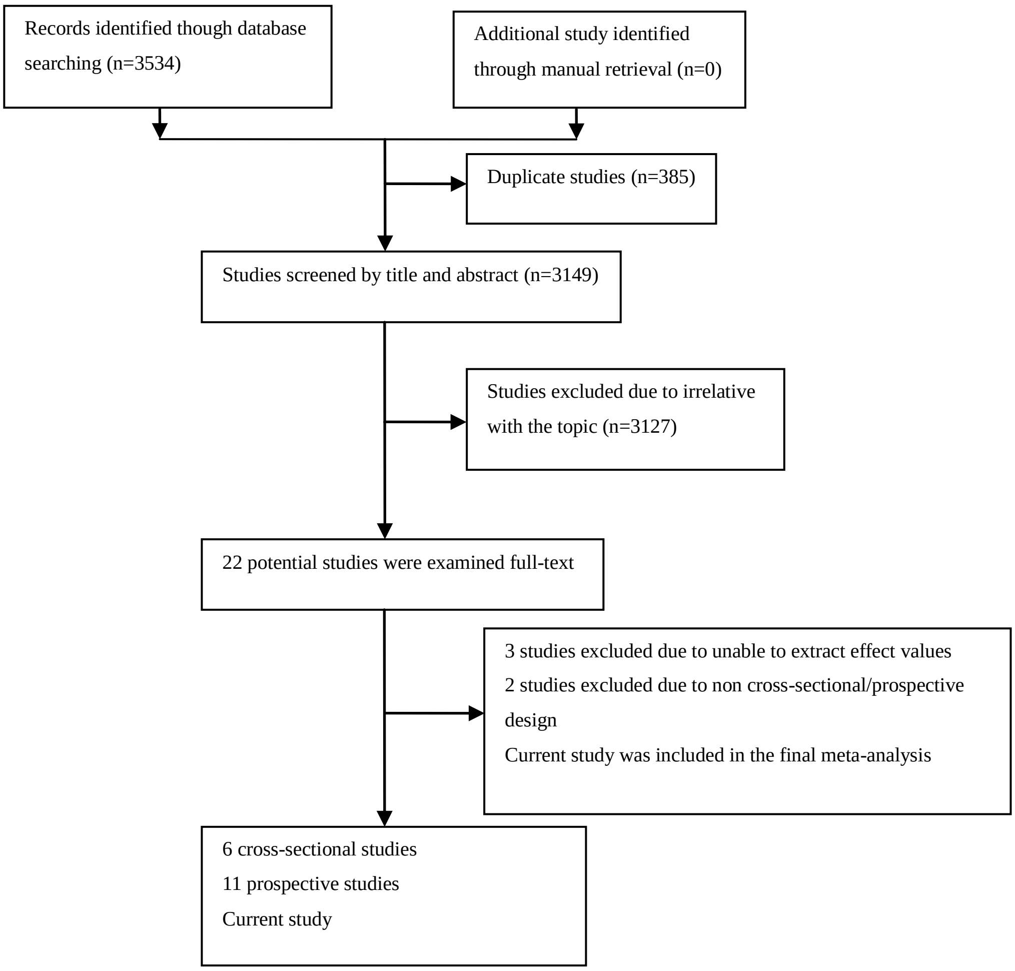

Supplementary Figure S1 Flow diagram of included studies for the meta-analysis of resting heart rate and diabetes risk

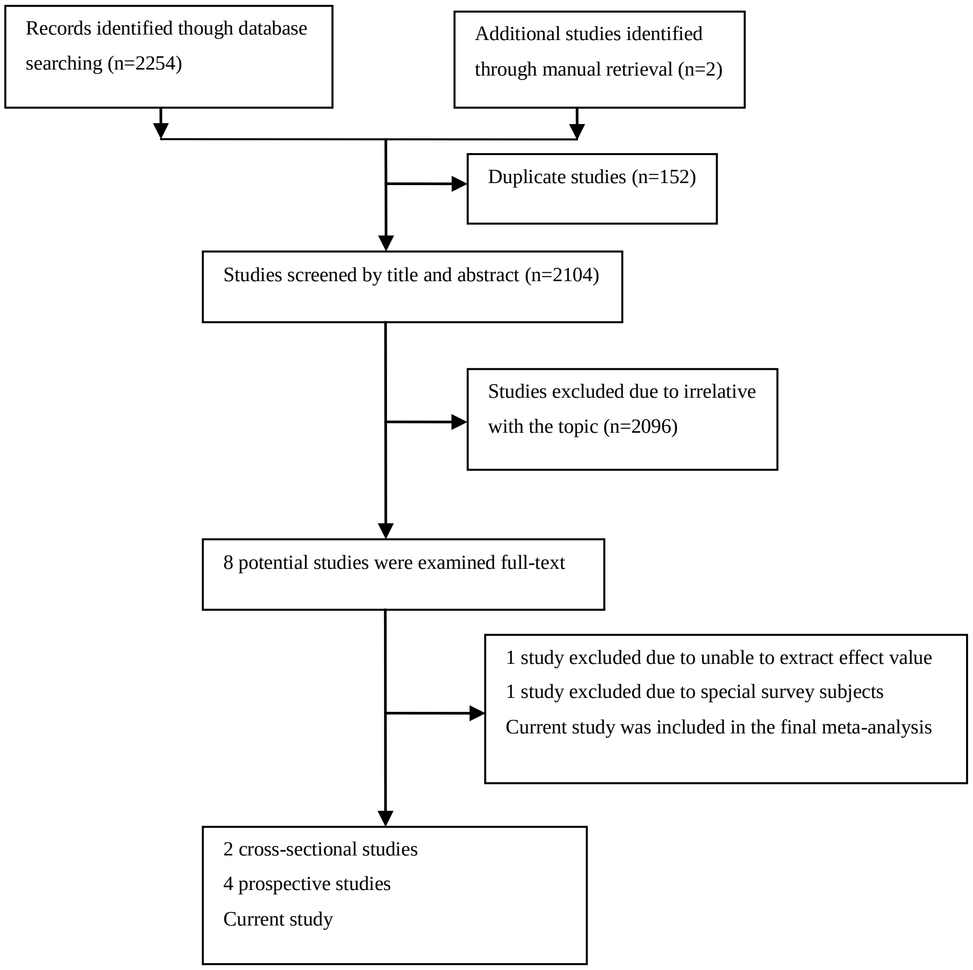

Supplementary Figure S2 Flow diagram of included studies for the meta-analysis of pulse pressure and diabetes risk

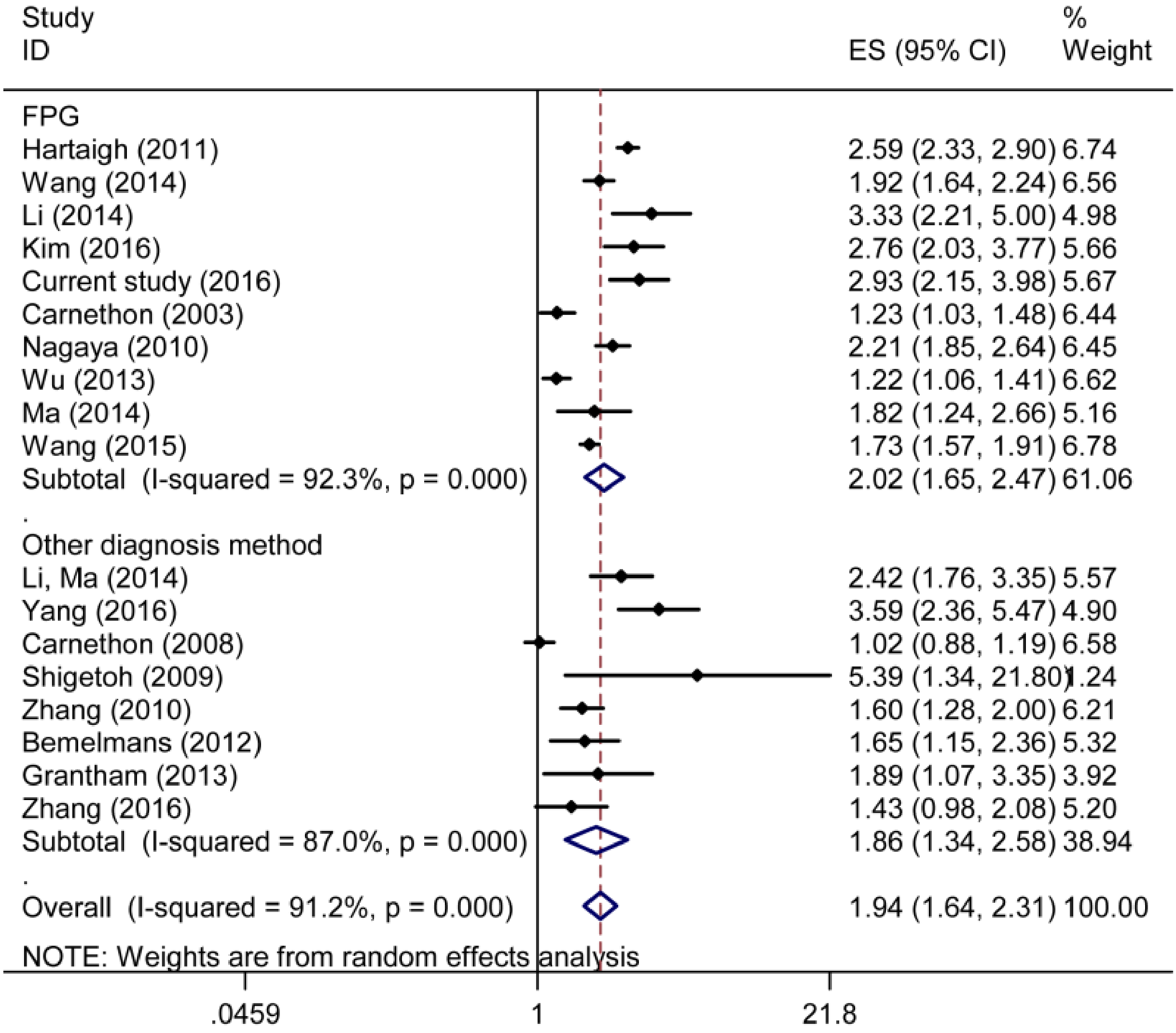

Supplementary Figure S3 Forest plot of the diagnosis criteria and diabetes risk for resting heart rate

Other diagnosis method: FPG/OGTT; FPG/HbA1c; in-person follow-up interviews; questionnaire /physicians validation. FPG: fasting plasma glucose; OGTT: oral glucose tolerance test.

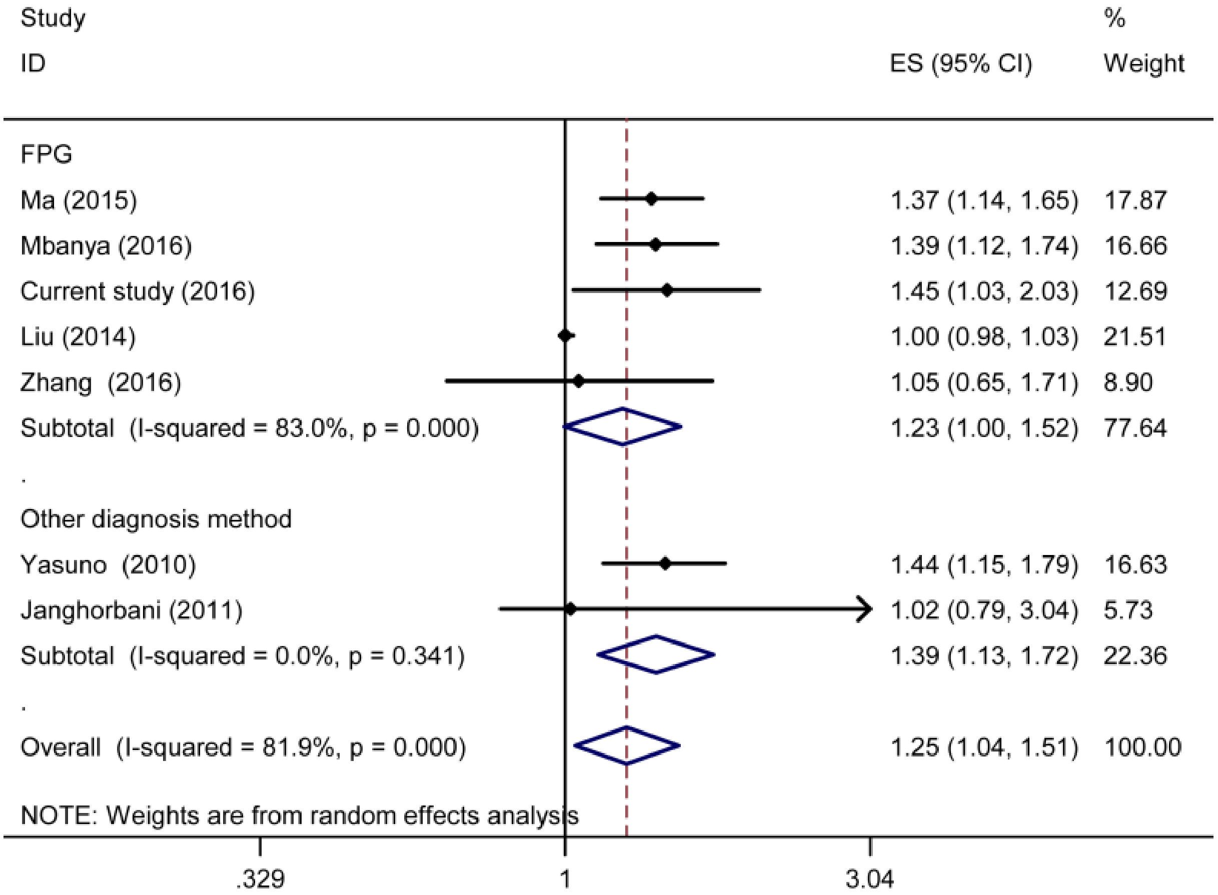

Supplementary Figure S4 Forest plot of the diagnosis criteria and diabetes risk for pulse pressure

Other diagnosis method: FPG/OGTT; individual case report. FPG: fasting plasma glucose; OGTT: oral glucose tolerance test.

First author (published year)	Country	Study name	Study design	Survey time	Sample size (male/female)	Age range (years)	Resting heart rate stratify method	Diagnosis method	Outcome	Case size (male/female)	Adjusted effect size
Hartaigh(2011)[26]	China	The Guangzhou Biobank Cohort Study	Cross-sectional	2003-2006	n=29,835<br>(8,240/21,595)	NA	High: ≥83	FPG	T2DM	n=3,777	2.59(2.33-2.90)
Li, Ma(2014)[27]	China	The Jiading community study in Shanghai	Cross-sectional	2010	n=2,233<br>(931/1,302)	>40	Tertiles	FPG/OGTT	T2DM	n=337<br>(163/174)	2.42(1.76-3.35)
Wang(2014)[28]	China	Medical information from Xiangcheng district in Suzhou	Cross-sectional	NA	n=8,954<br>(3,984/4,970)	>18	Fast: ≥85	FPG	Diabetes	NA	1.918(1.642-2.241)
Li(2014)[14]	China	The rural district of Luoyang City	Cross-sectional	2007-2008	n=16,636<br>(6,533/10,103)	35-78	Four categories	FPG	Undiagnosed T2DM	n=814<br>(283/531)	Male:3.66(2.09-6.37)<br>Female:2.98(1.64-5.42)
Yang(2016)[29]	Korea	The fifth Korea National Health and Nutrition Examination Survey (KNHANES)	Cross-sectional	2010-2012	n=18,640<br>(7,942/10,698)	>18	Five categories	FPG/HbA1c	Diabetes	NA	Male:3.85(2.14-6.90)<br>Female:3.34(1.83-6.10)
Kim(2016)[30]	Korea	The National Health Insurance Corporation (NHIC) examinations	Cross-sectional	2007-2010	n=5,124,<br>(904/4,220)	NA	Quartiles	FPG	T2DM	n=442	2.76(2.03-3.77)
Current study (2016)	China	The Rural Diabetes, Obesity and Lifestyle (RuralDiab) Study	Cross-sectional	2013-2015	n=8,276<br>(2,981/5,295)	35-74	Quartiles	FPG	Undiagnosed T2DM	n=383<br>(156/227)	Total:2.93(2.15-3.98)<br>Male:4.69(2.58-8.55)<br>Female:2.45(1.70-3.53)
Carnethon (2003)[8]	USA	The Atherosclerosis Risk In Communities (ARIC) Study	Cohort<br>(8.3years)	1987-1998	n=8,185	45-64	Quartiles	FPG	Diabetes	n=1,063	1.23(1.03-1.48)
Carnethon(2008)[9]	USA	The Chicago Heart Association Detection Project in Industry	Cohort	Baseline:1967-1973<br>Follow-up:1992-2002	n=14,992	35-64	Quartiles	50-g oral glucose load	Diabetes	n=1,877	1.02(0.88-1.19)
Shigetoh(2009)[11]	Japan	A cohort of the Seven Countries Study	Cohort<br>(20years)	1979-1999	n=614<br>(224/ 390)	>20	Four categories	FPG/HbA1c	Obesity and Diabetes	NA	5.39(1.34-21.8)
Nagaya(2010)[10]	Japan	A follow-up study of middle-aged healthy Japanese population	Cohort(male/ female: 7.4/7.1 years)	Baseline: 1988-1991<br>Follow-up:2001	n=25,196<br>(16,828/ 8,368)	30-59	Quartiles	FPG	Diabetes	n=1,093<br>(869/224)	Male:2.26(1.84-2.78)<br>Female:2.06(1.43-2.96)
Zhang(2010)[12]	China	Shanghai Women's Health Study(SWHS)	Cohort<br>(4.9 years)	Baseline:2000-2002<br>Follow-up: every two years	n=47,571	40-70	Five categories	In-person follow-up Interviews	T2DM	n=849	1.60(1.28-2.00)
Bemelmans (2012)[31]	The Netherlands	The second manifestations of arterial disease (SMART) study	Cohort<br>(5.5 years)	1996-2010	n=3,646	18-80	Quartiles	Questionnaire /physicians validation	T2DM	n=289	1.65(1.15-2.36)
Grantham (2013)[7]	Australia	The Australian Diabetes Obesity and Lifestyle Study	Cohort<br>(over 5 years)	Baseline:1999-2000<br>Follow-up:2004-2005	n=5,817<br>(2,628/3,189)	≥25	Four categories	FPG/OGTT	Diabetes	n=221<br>(113/108)	Total:1.89(1.07-3.35)<br>Male:2.02(0.93-4.39)<br>Female:1.72(0.72-4.12)
Wu(2013)[32]	China	Kailuan prospective study	Cohort<br>(3.95±0.51 years)	Baseline:2006-2007<br>Follow-up:2008-2009<br>2010-2011	n=29,910<br>(21,850/8,060)	45.91±11.47	Quartiles	FPG	New-onset diabetes	n=1,665	Total: 1.22(1.06-1.41)<br>Male:1.27(1.09-1.49)<br>Female:1.16(0.81-1.67)
Ma(2014)[33]	China	Medical information from Xiangcheng district in Suzhou	Cohort<br>(2 years)	NA	n=1,874<br>(861/1,013)	≥60	Fast: ≥85	FPG	T2DM	n=132<br>(62/70)	1.815(1.238-2.661)
Wang(2015)[13]	China	Kailuan prospective study	Cohort<br>(4 years)	Baseline:2006-2007<br>Follow-up: every two years	n=73,357<br>(57,719/15,638)	Male：50.3±12.5<br>Female：47.2±11.3	Quintile	FPG	Diabetes	n=4,649	Total:1.73(1.57-1.91)<br>Male:1.76(1.58-1.95)<br>Female:1.62(1.25-2.11)
Zhang(2016)[34]	China	Information from rural district in Inner Mongolia	Cohort<br>(10 years)	Baseline:2002-2003<br>Follow-up:2013	n=1,729<br>(659/1,070)	≥20	Tertile	FPG/OGTT	T2DM	n=155	1.43(0.98-2.08)

Supplementary Table S1 Information of studies included in meta-analysis of resting heart rate and diabetes risk. FPG: fasting plasma glucose; HbA1c: glycosylated hemoglobin A1c; OGTT: oral glucose tolerance test; NA: not available.

First author (published year)	Country	Study name	Study design	Survey time	Sample size (male/female)	Age range (ages)	Pulse pressure stratify method	Diagnosis method	Outcome	Case size (male/female)	Adjusted effect size
Ma(2015)[20]	China	Medical information from Xiangcheng district in Suzhou	Cross-sectional	2014	n=8,960 (3,985/4,975)	>18	Lower:<20 Normal:20-60 Higher:> 60	FPG	Diabetes	NA	1.37(1.14-1.65)
Mbanya(2016)[21]	Cameroon	The Cameroon Burden of Diabetes (CAMBoD) 2006–2008 community-based survey	Cross-sectional	2006	n=9,018 (3,544/5,474)	15-99	Quintile	FPG	Diabetes	n=405 (160/245)	1.39 (1.12–1.74)
Current study(2016)	China	The Rural Diabetes, Obesity and Lifestyle (RuralDiab) study	Cross-sectional	2013-2015	n=8,276 (2,981/5,295)	35-74	Quartile	FPG	Undiagnosed T2DM	n=383 (156/227)	Total : 1.45(1.03-2.03) Male: 0.86(0.51-1.44) Female: 1.69(1.07-2.67)
Yasuno(2010)[22]	Japan	The Candesartan Antihypertensive Survival Evaluation in Japan (CASE-J) trial	Cohort (3.3±0.8 years)	Baseline:2001.9-2002.12 Follow-up: every six months until 2005.9	n=2,685 (1,471/1,214)	20-84	Per 1SD increase	Individual case report	New-onset diabetes	n=97 (65/32)	1.44(1.15–1.79)
Janghorbani(2011)[23]	Iran	The Isfahan Diabetes Prevention Study (IDPS)	Cohort (2.3 years)	Baseline:2003-2005 Follow-up:2008	n=701 (150/551)	20-70	Quartile	FPG/OGTT	T2DM	n=72 (11/61)	1.02(0.79-3.04)
Liu(2014)[24]	China	The Chinese Multi-provincial Cohort Study (CMCS)	Cohort (15 years)	Baseline:1992 Follow-up:2007	n=687 (399/288)	35-64	NA	FPG	Diabetes	n=74 (48/26)	1.003 (0.981–1.026)
Zhang(2016)[25]	China	Information from a rural area of Henan province	Cohort (6 years)	Baseline:2007-2008 Follow-up:2013-2014	n=12,272 (4,664/7,608)	≥18	Normal：20-60 High：>60	FPG	T2DM	n=775 (296/479)	Male: 0.793(0.498-1.262) Female: 1.304(0.979-1.736)

Supplementary Table S2 Information of studies included in meta-analysis of pulse pressure and diabetes risk. FPG: fasting plasma glucose; OGTT: oral glucose tolerance test; NA: not available.
